# A nomogram for predicting sepsis-associated delirium: a retrospective study in MIMIC III

**DOI:** 10.1186/s12911-023-02282-5

**Published:** 2023-09-15

**Authors:** Qiong Gu, Shucong Yang, DanTing Fei, Yuting Lu, Huijie Yu

**Affiliations:** Department of Emergency Medicine, The First Hospital of Jiaxing/Affiliated Hospital of Jiaxing University, Jiaxing, 314001 China

**Keywords:** Sepsis, Sepsis-associated delirium, Delirium, Nomogram

## Abstract

**Objective:**

To develop a nomogram for predicting the occurrence of sepsis-associated delirium (SAD).

**Materials and methods:**

Data from a total of 642 patients were retrieved from the Medical Information Mart for Intensive Care (MIMIC III) database to build a prediction model. Multivariate logistic regression was performed to identify independent predictors and establish a nomogram to predict the occurrence of SAD. The performance of the nomogram was assessed in terms of discrimination and calibration by bootstrapping with 1000 resamples.

**Results:**

Multivariate logistic regression identified 4 independent predictors for patients with SAD, including Sepsis-related Organ Failure Assessment(SOFA) (p = 0.004; OR: 1.131; 95% CI 1.040 to 1.231), mechanical ventilation (P < 0.001; OR: 3.710; 95% CI 2.452 to 5.676), phosphate (P = 0.047; OR: 1.165; 95% CI 1.003 to 1.358), and lactate (P = 0.023; OR: 1.135; 95% CI 1.021 to 1.270) within 24 h of intensive care unit (ICU) admission. The area under the curve (AUC) of the predictive model was 0.742 in the training set and 0.713 in the validation set. The Hosmer − Lemeshow test showed that the model was a good fit (p = 0.471). The calibration curve of the predictive model was close to the ideal curve in both the training and validation sets. The DCA curve also showed that the predictive nomogram was clinically useful.

**Conclusion:**

We constructed a nomogram for the personalized prediction of delirium in sepsis patients, which had satisfactory performance and clinical utility and thus could help clinicians identify patients with SAD in a timely manner, perform early intervention, and improve their neurological outcomes.

**Supplementary Information:**

The online version contains supplementary material available at 10.1186/s12911-023-02282-5.

## Introduction

Although sepsis has been well researched over the past three decades, it is still a major cause of morbidity and mortality worldwide, thus causing a great financial burden to families and societies in general [1, 2]. In recent years, sepsis-related complications have also received increasing attention, especially sepsis-associated encephalopathy (SAE), which has been reported in up to 70% of patients with severe systemic infection [3]. Among the clinical manifestations of SAE, delirium is most common, accounting for 49%, while coma, focal neurological signs, and seizure account for 46%, 18%, and 10%, respectively [4]. Delirium is also commonly encountered in ICUs. According to the diagnostic criteria for delirium by the American Psychiatric Association’s Fifth Edition of the Diagnostic and Statistical Manual of Mental Disorders (DSM-5) [5], all states of altered arousal, except coma, can be defined as delirium. The occurrence of delirium and coma are usually indicative of acute brain dysfunction [6]. A previous multicenter study reported that 307 of 1,333 patients (23%) with severe sepsis had an acute alteration in mental status [7]. Delirium associated with sepsis or other causes was found to be an independent predictor of the 6-month mortality rate in a prospective cohort study [8]. Moreover, sepsis-associated delirium (SAD) is a serious neurological complication of sepsis. The pathophysiology of SAD includes systemic inflammation, which can predispose patients to blood‒brain barrier disruption, resulting in peripheral leukocyte infiltration into the central nervous system [9–11]. The resultant neuroinflammation promotes a state of cholinergic failure, predisposing patients to delirium [12].

Delirium diagnosis is usually based on the absence of direct central nervous system infection, multiorgan failure, traumatic brain injury, fat embolism, and ingestion of illicit drugs [13]. In practice, the diagnostic approach varies according to sedation. Since the development and widespread use of validated sedation screening tools, the clinical data for SAD have steadily accumulated. The RASS/CAM-ICU is a common and well-validated screening tool for delirium. Currently, there are still no specific diagnostic blood tests, electrophysiological tests, or imaging tests, as well as no specific treatments for SAD.

The duration of delirium in the ICU is associated with long-term functional disability and cognitive impairment [14–16]. Thus, early diagnosis and initiation of rehabilitation for brain injury are crucial for the survival and prognosis of patients with sepsis. The aim of this study was to create a prediction model with potential risk factors for the early identification of SAD by a retrospective analysis of a large clinical database.

## Materials and methods

### Study design and participants

#### Data resource

MIMIC-III (medical information mart for intensive care) is a multiparameter critical care database with open access that contains comprehensive and high-quality data of well-defined and characterized ICU patients admitted to ICUs at the Beth Israel Deaconess Medical Center between 2001 and 2012. The National Institutes of Health’s web-based course has been completed, and the certification (researcher certificate number: 46,429,516) was acquired.

#### Patient population

**Fig. 1:**
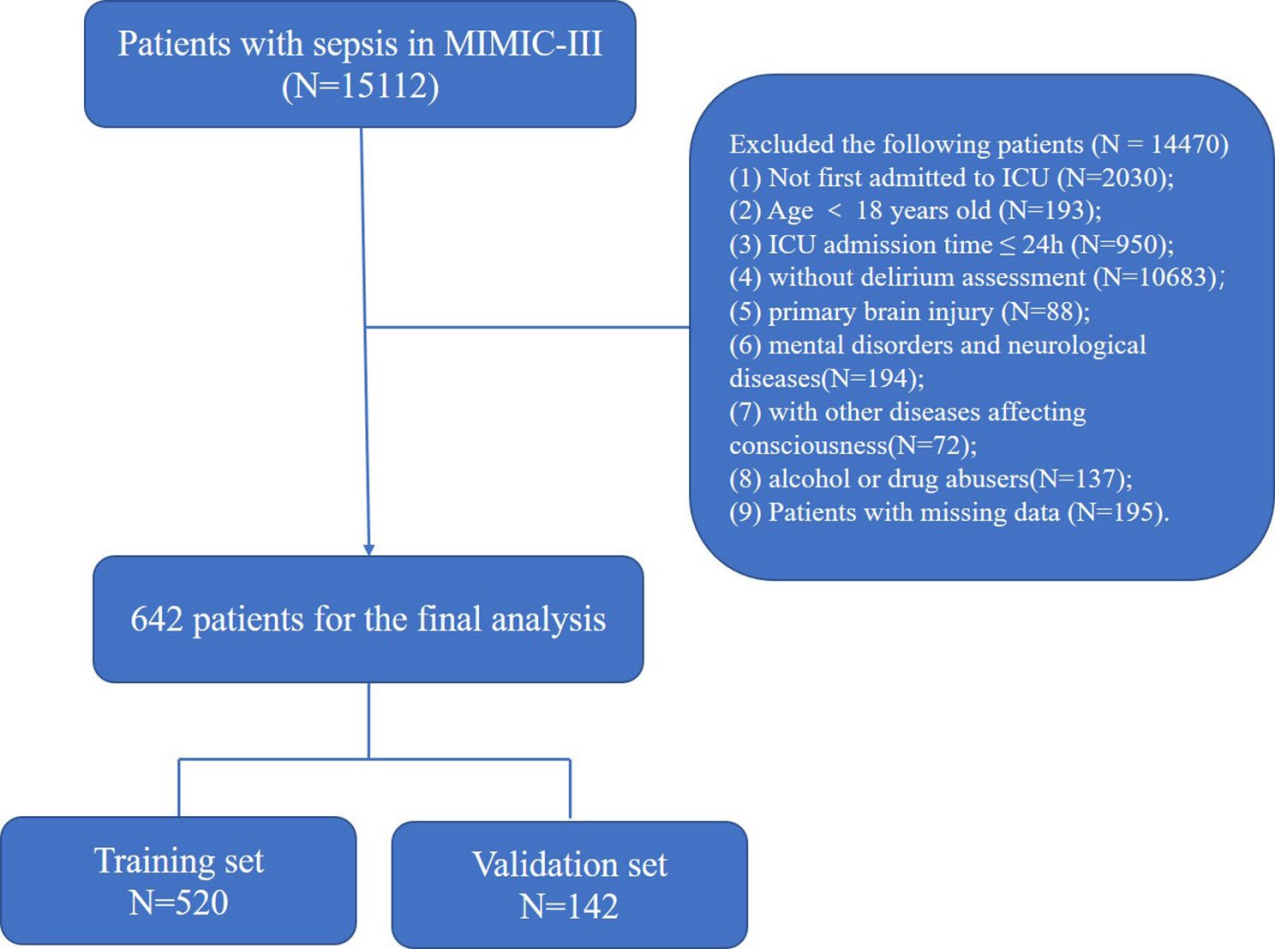
Flow chart of the patient selection (MIMIC III, Medical Information Mart for Intensive Care III).

We extracted patient information from the MIMIC III database according to the following inclusion criteria: (1) first admitted to the ICU; (2) age ≥ 18 years old; (3) diagnosed with sepsis; (4) ICU admission time ≥ 24 h; and (5) delirium assessment. The diagnosis of sepsis was based on the definition of sepsis 3.0, which includes patients with documented or suspected infection and SOFA score ≥ 2 points. Infection was identified with microbiological culture results. Sepsis-associated delirium in our study was defined as sepsis patients with a positive assessment of CAM-ICU and delirium assessment during their stay in ICUs. The patient enrollment process of the study is illustrated in Fig. 1.

#### Data collection

All research data were extracted from the MIMIC-III by Structured query language (SQL) with Navicat Premium, and they included (1) patients’ demographics; (2) the first-day vital sign, laboratory data, and respiratory support; (3) severity score including GCS, SOFA, and SAPII; (4) other comorbidities in ICU; and (5) details of admission including length of stay in the hospital, length of stay in ICU, and morality.

#### Sepsis-associated delirium

The Confusion Assessment Method for ICU Patients (CAM-ICU) is a useful screening tool for identifying delirium, the results of which were extracted from the MIMIC-III database on patients’ admission to the ICU. Patients who were unable to undergo the assessment were excluded. Sepsis patients whose delirium assessment was positive were defined as having delirium. The definition of sepsis-associated delirium was based on the delirium assessment. Patients with delirium related to drug or alcohol abuse, neurological diseases, mental disorders, and other diseases were excluded.

### Statistical analysis

The study population was divided into two groups according to their delirium assessment. The distribution of data was analyzed by the Shapiro–Wilk test. Parameters with continuous data were expressed as the mean ± standard deviation or the median (interquartile range, IQR); categorical variables were expressed as frequency and rate (%). A nonparametric test (Mann–Whitney U test or Kruskal–Wallis test) was used for data with a nonnormal distribution or heterogeneity of variances. Categorical data were compared using the Pearson chi-square test. All statistical analyses were performed by R software with the tableone, rms, pROC, dca, and rdma packages.

In our research, variables that were significant (P < 0.05) in the univariate analysis were subsequently entered into the multivariate logistic regression model. It is worth mentioning that comorbidity (P < 0.05) was not included as a risk factor because we extracted the data according to patient diagnoses, and the diagnoses from MIMIC-III were discharge diagnoses, which may not be available at the time of admission. Odds ratios (ORs) and 95% confidence intervals (95% CIs) were calculated [17].

To develop the prediction nomogram, patients were randomly distributed into training and validation sets at a ratio of 8:2. Risk factors for the nomogram were chosen from the multivariate logistic regression where the variables were P < 0.05. For convenience of clinical decision-making, we considered few variables into the prediction model. The performance of the nomogram was evaluated in both the training and validation sets by the area under the curve of the receiver operating characteristic (AUROC). Additionally, the calibration curve and Hosmer‒Lemeshow test (HL test) were used to evaluate the accuracy by comparing the nomogram [18]. Decision curve analysis (DCA) was performed to evaluate the clinical use of the nomogram [19].

R software (version 4.1.3, R Foundation for Statistical Computing, Vienna, Austria) was used for all statistical analyses. A P value < 0.05 was considered statistically significant.

## Results

### Participants

First, patients with sepsis were selected according to the definition of sepsis 3.0 and excluded based on the diagnosis listed in the exclusion criteria. Then, we retrieved some laboratory tests and the delirium assessment for eligible patients, excluding those who were unable to participate in the assessment. Additionally, patients with missing data were excluded. Finally, 642 sepsis patients with delirium assessment were included in the analysis. Characteristic baseline and details at admission to the ICU are shown in Table 1. Sepsis patients with other neurological diseases were more likely to suffer from delirium, while the incidence of delirium was lower with cardiovascular diseases. It is obvious that patients with SAD have a worse prognosis, and patients with SAD had a longer stay in both the hospital and ICU (P < 0.001). Additionally, they had a higher rate of 28-day mortality (P < 0.001). The first-day means revealed no significant differences in vital signs between the two groups. Patients suffering from SAD had higher SAPS II and SOFA scores but lower GCS scores.

**Table 1:** Characteristics at baseline between the non-SAD and SAD groups

		Total (n = 642)	Non-SAD (n = 414)	SAD (n = 228)	P-value
**Age, years**		68.72 [56.87, 79.14]	69.13 [57.12, 79.54]	66.56 [56.77, 77.70]	0.483
**Gender (n, %)**	Male	326 (50.8)	200 (48.3)	126 (55.3)	0.109
	Female	316 (49.2)	214 (51.7)	102 (44.7)	
**Ethnicity (n, %)**	White	429 (66.8)	287 (69.3)	142 (62.3)	0.001
	Asian	18 (2.8)	14 (3.4)	4 (1.8)	
	Black	83 (12.9)	53 (12.8)	30 (13.2)	
	Hispanic or Latino	26 (4.0)	21 (5.1)	5 (2.2)	
	Others	86 (13.4)	39 (9.4)	47 (20.6)	
**Admission type (n, %)**	Elective	42 (6.5)	27 (6.5)	15 (6.6)	0.993
	Emergency	594 (92.5)	383 (92.5)	211 (92.5)	
	Urgent	6 (0.9)	4 (1.0)	2 (0.9)	
**severe score**	SAPSII	28.00 [20.00, 38.00]	26.00 [19.00, 34.00]	32.00 [24.00, 42.00]	< 0.001
	SOFA	5.00 [4.00, 8.00]	5.00 [3.00, 7.00]	7.00 [4.00, 10.00]	< 0.001
	GCS	15.00 [14.00, 15.00]	15.00 [14.00, 15.00]	14.00 [13.00, 15.00]	0.004
**Hospital length of stay, days**		10.32 [6.48, 17.89]	8.29 [5.81, 13.88]	15.22 [9.71, 22.14]	< 0.001
**ICU length of stay, days**		3.65 [2.00, 7.56]	2.75 [1.71, 4.62]	7.61 [3.80, 13.82]	< 0.001
**Mechanical ventilation (n, %)**		322 (50.2)	158 (38.2)	164 (71.9)	< 0.001
**28-day mortality in hospital(n, %)**		124 (19.3)	60 (14.5)	64 (28.1)	< 0.001
**Vital signs**	Mean heartrate (min-1)	88.96 [76.46, 101.25]	88.77 [76.11, 100.31]	90.19 [77.09, 102.98]	0.174
	Mean arterial pressure (mmHg)	71.89 [66.18, 79.35]	71.61 [65.67, 78.66]	73.38 [67.31, 80.25]	0.109
	Mean respiratory rate (min-1)	19.70 [16.97, 22.72]	19.45 [16.78, 22.54]	20.14 [17.22, 22.98]	0.096
	Mean temperature (°C)	36.83 [36.50, 37.21]	36.83 [36.50, 37.20]	36.85 [36.50, 37.30]	0.359
	Mean SpO2 (%)	97.28 [95.93, 98.54]	97.16 [95.77, 98.48]	97.56 [96.30, 98.67]	0.079
**Laboratory tests**	Blood glucose (mg/dl)	135.88 [111.21, 166.50]	134.88 [111.10, 164.14]	139.00 [112.24, 174.52]	0.209
	WBC (K/uL)	11.80 [7.40, 16.60]	11.30 [7.20, 15.88]	12.70 [8.05, 17.83]	0.017
	RBC (K/uL)	3.54 [3.04, 4.11]	3.54 [3.01, 4.07]	3.53 [3.08, 4.18]	0.402
	Hemoglobin (g/dL)	10.60 [9.10, 12.50]	10.55 [9.10, 12.40]	10.75 [9.20, 12.60]	0.507
	Platelet (K/uL)	211.00 [142.00, 289.75]	212.00 [144.00, 288.25]	209.50 [139.50, 293.25]	0.774
	HCT (%)	32.65 [28.22, 37.60]	32.40 [27.72, 37.30]	33.45 [28.58, 38.52]	0.076
	Creatinine (mg/dl)	1.30 [0.90, 2.20]	1.30 [0.90, 2.00]	1.40 [0.90, 2.40]	0.218
	Phosphate (mg/dl)	3.60 [2.70, 4.60]	3.50 [2.70, 4.40]	4.00 [3.00, 5.00]	< 0.001
	BUN (mg/dl)	27.00 [17.00, 46.00]	27.00 [17.00, 45.00]	27.00 [17.75, 49.25]	0.413
	Sodium (K/uL)	137.00 [134.00, 140.00]	137.00 [133.00, 140.00]	137.00 [134.00, 140.00]	0.146
	Potassium (K/uL)	4.20 [3.80, 4.80]	4.20 [3.70, 4.70]	4.40 [3.90, 4.82]	0.016
	Lactate (mmol/L)	1.80 [1.30, 2.80]	1.70 [1.22, 2.50]	1.90 [1.30, 3.20]	0.015
**Comorbodity (n, %)**	Cardiovascular diseases	410 (63.9)	252 (60.9)	158 (69.3)	0.041
	Peripheral vascular	80 (12.5)	47 (11.4)	33 (14.5)	0.307
	Hypertension	419 (65.3)	264 (63.8)	155 (68.0)	0.324
	Other neurological diseases	80 (12.5)	36 (8.7)	44 (19.3)	< 0.001
	Chronic pulmonary	220 (34.3)	133 (32.1)	87 (38.2)	0.146
	Renel failure	199 (31.0)	129 (31.2)	70 (30.7)	0.975
	Liver failure	98 (15.3)	58 (14.0)	40 (17.5)	0.282
	DM	242 (37.7)	164 (39.6)	78 (34.2)	0.205

In laboratory tests, patient ethnicity, phosphate, potassium, lactate, and WBC significantly differed between the SAD and non-SAD groups. The characteristics at baseline between the training and validation groups were divided by an 8:2 ratio (Additional file 2: Table S1). There were no significant differences in other clinical factors between the two datasets (P = 0.182 to 0.941) except for age (P = 0.005).

### Screening for predictive factors

First, we conducted a multivariate logistic regression analysis in the training group with a P value < 0.05 in the baseline comparison, which identified 4 factors as independent predictors of SAD, per the following results: SOFA (p = 0.004; OR: 1.131; 95% CI 1.040 to 1.231), mechanical ventilation (P < 0.001; OR: 3.710; 95% CI 2.452 to 5.676), phosphate (P = 0.047; OR: 1.165; 95% CI 1.003 to 1.358), and lactate (P = 0.023; OR: 1.135; 95% CI 1.021 to 1.270). These data are shown in Table 2.

**Table 2:** Multivariate logistic analysis of the training group

	OR	CI 2.5%	97.50%	P Value
SAPSII	0.996	0.972	1.020	0.736
SOFA	1.131	1.040	1.232	0.004
GCS	0.924	0.850	1.002	0.059
Ethnicity	0.917	0.769	1.096	0.336
Mechanical ventilation	3.710	2.452	5.676	< 0.001
WBC	1.021	1.003	1.042	0.053
Phosphate	1.165	1.003	1.358	0.047
Lactate	1.135	1.021	1.270	0.023
Potassium	1.035	0.807	1.320	0.782

### Risk prediction nomogram development

**Fig. 2:**
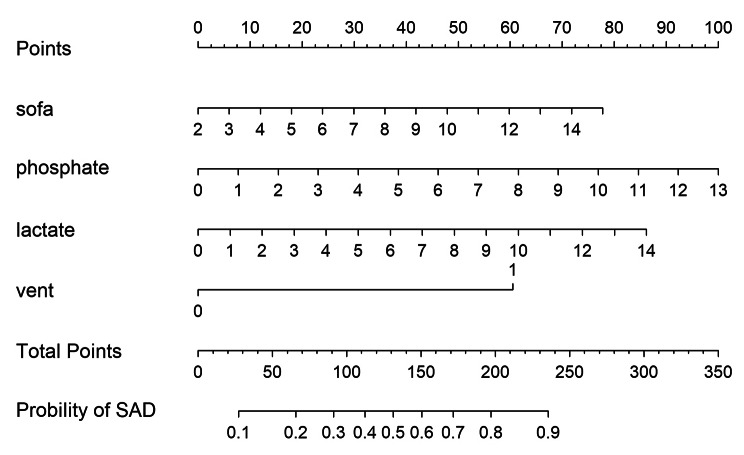
Nomogram for sepsis-associated delirium

Four predictors were included in the final multivariable logistic model, i.e., SOFA (OR: 0.97; 95% CI 0.96 to 1.01), mechanical ventilation (OR: 4.18; 95% CI 2.67 to 6.66), first phosphate (OR: 1.19; 95% CI 1.01 to 1.40), and first lactate (OR: 1.1; 95% CI 1.02 to 1.29). A nomogram for predicting the probability of SAD was constructed by the logistic analysis model (Fig. 2). For each patient, total high points indicated a higher risk of SAD. For example, if the SOFA score of a patient with sepsis was 3 points, phosphate was 5.5 mg/dl, lactate was 4 mmol/L, and the patient received no mechanical ventilation, the corresponding scores were 5, 41, 22, and 0, respectively. The total score was 68, which indicated that the estimated risk of SAD was 0.21 for this case. In addition, the Hosmer‒Lemeshow test for the training set was 0.326, and the Hosmer‒Lemeshow test for the validation set was 0.471 with the prediction probability of the training set, which demonstrated that the model was a good fit. Additionally, we conducted the Hosmer‒Lemeshow test for the validation set separately (P = 0.146).

### Predictive accuracy and net benefit of the nomogram

**Fig. 3:**
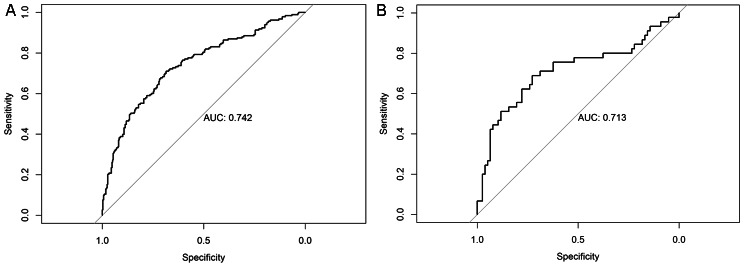
ROC curves for the nomogram. **Left**: Training group; **Right**: Validation group

**Fig. 4:**
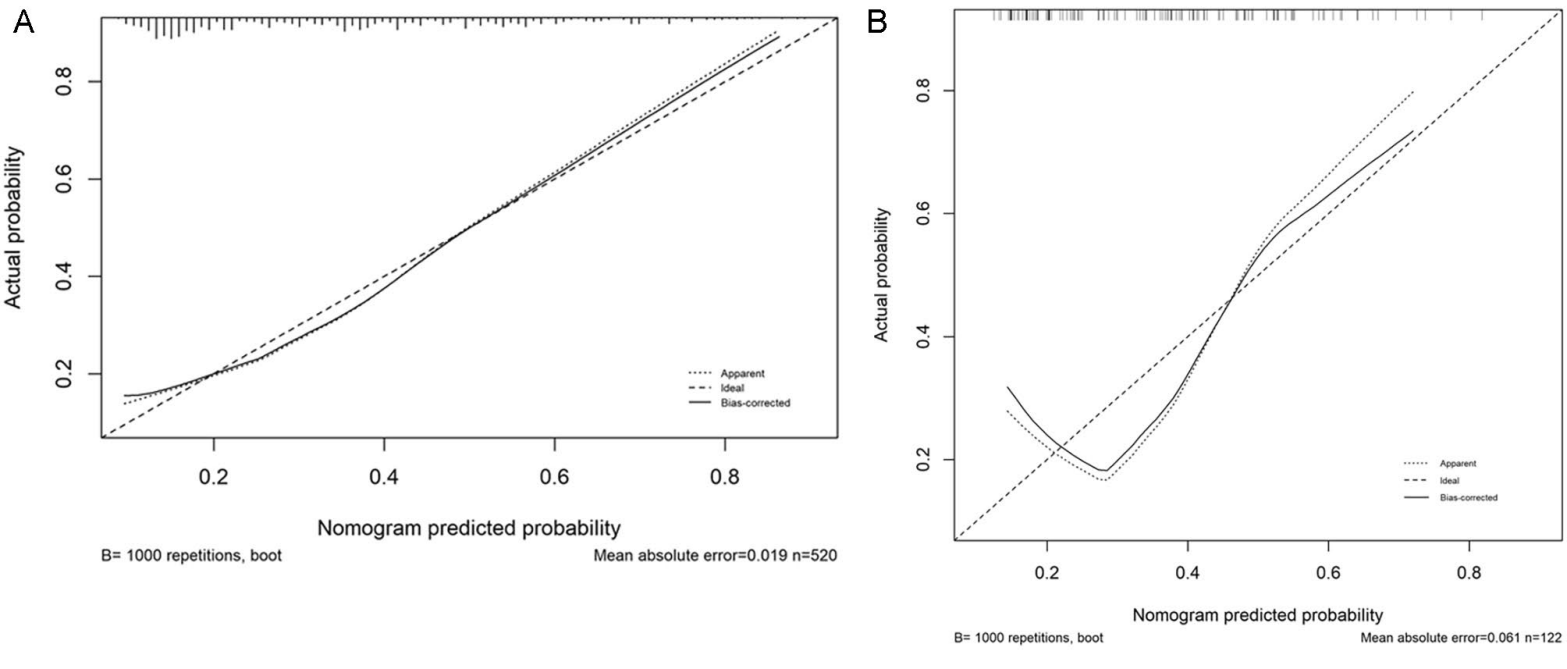
Calibration curve for predicting the probability of SAD. **Left**: Training group; **Right**: Validation group

**Fig. 5:**
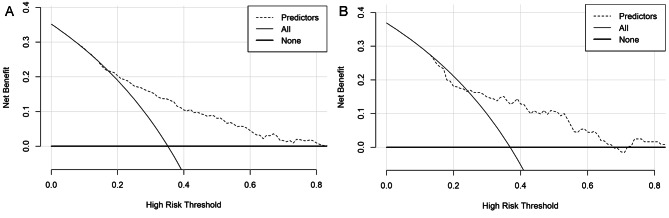
Decision curve analysis in the prediction of SAD. **Left**: Training group; **Right**: Validation group

The AUC of the training cohort was 0.742 (Fig. 3), and that of the validation cohort was 0.713 (Fig. 3). The calibration curve was close to the ideal diagonal line (Fig. 4). Additionally, DCA showed a significantly better net benefit in the predictive model (Fig. 5). Additionally, the calibration curve and DCA of the validation set also performed well. (Figures 4 and 5). The threshold probability range for the training set was 0.09–0.97, and that for the validation set was 0.14–0.46.

## Discussion

While it was previously thought that sepsis-associated encephalopathy is entirely reversible, recent studies have reported that infectious disease and systemic inflammation are risk factors for delirium, cognitive impairment, and neurodegenerative diseases [20–22]. Moreover, even among people who survived sepsis, a substantial proportion developed diffuse long-term cognitive deficits and had a higher risk of developing dementia [23].

Sepsis-associated delirium remains underdiagnosed, probably as a consequence of the lack of attention from treating physicians [24]. Therefore, it is important to increase the awareness of clinicians about this problem so that they can objectively estimate the risks/benefits of early medical intervention in patients with sepsis-associated delirium. Usually, the diagnosis of SAD occurs primarily in states of confusion and emotional dysfunction. Currently, the Confusion Assessment Method for the Intensive Care Unit (CAM-ICU) (sensitivity of 0.79 and specificity of 0.64) and the Intensive Care Delirium Screening Checklist (ICDSC) (sensitivity of 0.99 and specificity of 0.97) are the most valid and reliable tools for monitoring delirium in adult ICU patients [25–28].

The present study was based on the CAM-ICU and focused on predicting the model of SAD with patients’ SOFA scores, mechanical ventilation, first lactate, and first serum phosphate, which were used to calculate the probability of SAD and help clinicians identify patients with high risk and evaluate the treatment options to make better medical decisions.

The pathology of sepsis-associated delirium remains obscure. Systemic inflammation and endothelial activation frequently occur during sepsis and may augment cytokine transport across the blood‒brain barrier (BBB) [29]. Neuroinflammation activates microglia, which produce local proinflammatory cytokines and reactive oxygen species, and activates astrocytes [30], resulting in elevated S-100β levels [31], with the external manifestation of delirium. Previous studies have reported the associations between systemic biomarkers of inflammation (IL-6, IL-8, C-reactive protein) and S-100β with delirium occurrence [32]; however, we found no positive association between inflammation biomarkers and neurological outcomes, which may be due to the limited laboratory tests used in the present study.

The Sequential [Sepsis-related] Organ Failure Assessment (SOFA) score is a useful tool for identifying the risk of sepsis, which is associated with mortality when the score > = 2 points. The SOFA score is commonly regarded as a criterion for sepsis, while in some studies, it is used to predict and evaluate neurological outcomes [33, 34]. The SOFA score is a scoring system used to assess the severity of multiorgan dysfunction, which includes the evaluation of the central nervous system with the Glasgow (GCS) score. In the present study, the univariate analysis revealed that SOFA, GCS, and scores were higher in the SAD group. In the multivariate analysis, only SOFA score 0.05. There is a concern that patients with poor neurological status on admission rather than multiorgan dysfunction might have long-term neurological outcomes, which might deny the value of the SOFA score. A previous study that used the assessment of extracerebral SOFA (EC-SOFA) demonstrated that the EC-SOFA score and the change in the EC-SOFA score over 48 h were associated with neurological outcomes at 30 days [33].

Established sepsis and septic shock guidelines recommend limited continuous or intermittent sedation for mechanically ventilated sepsis patients [35]. In a prospective cohort study, delirium was independently associated with higher 6-month mortality and longer hospital stay in mechanically ventilated patients in the ICU [8]. In the present study, patients with sepsis-associated delirium had a higher proportion of mechanical ventilation (158 vs. 164, p < 0.001). This could be explained by the fact that ventilation control depends on a brainstem neuronal network, which orchestrates the activity of the motor neurons innervating the respiratory muscles. This network comprises the pontine respiratory group and dorsal and ventral respiratory groups in the medulla [36, 37]. Therefore, with neurologic or respiratory motor disorders, there is a higher risk of respiratory failure, which is a major cause of mechanical ventilation. However, in a study of combined protocols for sedation and interruption and mechanical ventilation weaning, that strategy did not prevent cognitive impairment in mechanically ventilated patients after 3 months or 1 year of follow-up [38].

Lactate is a product of anaerobic metabolism associated with the perfusion of tissue. In patients with sepsis, lactate elevation, as well as slow lactate clearance, was found to be related to worse clinical outcomes [39, 40]. We found that lactate was higher in SAD patients, which may be explained by the dysfunction of cerebral metabolism with the effect of metabolic acidosis. In their study, Sakpichaisakul et al. found lactate to be a predictor of neonatal encephalopathy severity [41] because of hypoxia in fetuses and infants. However, in a porcine cardiac arrest model, there were no significant differences between cerebral perfusion and lactate [42], i.e., lactate change would not affect the brain’s metabolism. Therefore, whether lactate is a meaningful predictive factor of SAD needs to be further validated.

Serum phosphate has only been reported in both sepsis and neurological diseases. To the best of our knowledge, this is the first study that mentioned the relationship between sepsis-associated delirium and serum phosphate. The vast majority of phosphate in the body is in the form of hydroxyapatite, such as in teeth and bone. The remaining phosphate is in the form of inorganic phosphate that is stored in extracellular fluids. Additionally, inorganic phosphate is a macronutrient for various cellular functions, including structure, energy production, metabolic pathways, and signaling [43].

A recent retrospective study that assessed the effect of serum phosphate on the prognosis of sepsis patients [44] found that even with the normal range of serum phosphate, a minor increase was related to a higher risk of in-hospital mortality, which was explained by subclinical vascular disease and lower muscle strength. Some studies have shown that hyperphosphatemia is related to cognitive impairment induced by vascular disease [45, 46]. A prospective study that included older men showed that higher serum phosphate was associated with a higher likelihood of poor executive function but not with impaired global cognitive function [47]. However, whether higher phosphate was a direct cause of neurological dysfunction or a prediction factor of disease severity remains unclear.

There are several limitations in the present study that should be noted. First, this is a retrospective observational study, and such studies have inherent biases. Second, the database included patient information from 2001 to 2012, and it was impossible to obtain some laboratory tests, such as PCT, CRP, and IL-6. Consequently, the prediction model could not include some potential risk factors. Additionally, this study was a single-center study, and the prediction model was validated using internal data from the same database. In future studies, data from the real world or other databases could be included to prove the accuracy of the prediction model. Third, CAM-ICU is suitable for conscious patients, which means our results cannot be applied to patients in a coma. Finally, there exist few prediction models for delirium, especially sepsis-associated delirium, which include serum phosphate. The association among delirium, serum phosphate and sepsis needs to be further studied and validated in the future.

## Conclusion

SOFA score, requiring mechanical ventilation, first lactate level, and first serum phosphate on ICU admission are factors that can predict the occurrence of delirium in sepsis patients. Here, we created a nomogram to provide clinicians with a visual and personalized tool for the early detection and identification of delirium in sepsis patients, which can result in earlier intervention and lower mortality rates related to delirium.

### Electronic supplementary material

Below is the link to the electronic supplementary material.

Additional file 1: ICD-9 codes for excluding diagnosis

Additional file 2: Table S1: characteristics at baseline between the training and validation groups were divided by the 8:2 ratio

## Data Availability

The data of our study were extracted from the MIMIC-III (Medical Information Mart for Intensive Care III) database (https://mimic.mit.edu/docs/iii/). The database is available for credentialed users on PhysioNet. However, the data and the code for the study are available from the corresponding author on reasonable request.

## References

[1] Fleischmann C, Thomas-Rueddel DO, Hartmann M, Hartog CS, Welte T, Heublein S, Dennler U, Reinhart K (2016). Hospital Incidence and Mortality Rates of Sepsis. Dtsch Arztebl Int.

[2] Fleischmann C, Scherag A, Adhikari NK, Hartog CS, Tsaganos T, Schlattmann P, Angus DC, Reinhart K (2016). International Forum of Acute Care T: Assessment of Global Incidence and Mortality of Hospital-treated Sepsis. Current estimates and Limitations. Am J Respir Crit Care Med.

[3] Gofton TE, Young GB (2012). Sepsis-associated encephalopathy. Nat Rev Neurol.

[4] Polito A, Eischwald F, Maho AL, Polito A, Azabou E, Annane D, Chretien F, Stevens RD, Carlier R, Sharshar T (2013). Pattern of brain injury in the acute setting of human septic shock. Crit Care.

[5] European Delirium A, American Delirium S (2014). The DSM-5 criteria, level of arousal and delirium diagnosis: inclusiveness is safer. BMC Med.

[6] Pandharipande PP, Pun BT, Herr DL, Maze M, Girard TD, Miller RR, Shintani AK, Thompson JL, Jackson JC, Deppen SA (2007). Effect of sedation with dexmedetomidine vs lorazepam on acute brain dysfunction in mechanically ventilated patients: the MENDS randomized controlled trial. JAMA.

[7] Sprung CL, Peduzzi PN, Shatney CH, Schein RM, Wilson MF, Sheagren JN, Hinshaw LB (1990). Impact of encephalopathy on mortality in the sepsis syndrome. The Veterans Administration systemic Sepsis Cooperative Study Group. Crit Care Med.

[8] Ely EW, Shintani A, Truman B, Speroff T, Gordon SM, Harrell FE, Inouye SK, Bernard GR, Dittus RS (2004). Delirium as a predictor of mortality in mechanically ventilated patients in the intensive care unit. JAMA.

[9] Bjornsson GL, Thorsteinsson L, Gudmundsson KO, Jonsson H, Gudmundsson S, Gudbjornsson B (2007). Inflammatory cytokines in relation to adrenal response following total hip replacement. Scand J Immunol.

[10] Hofer S, Bopp C, Hoerner C, Plaschke K, Faden RM, Martin E, Bardenheuer HJ, Weigand MA (2008). Injury of the blood brain barrier and up-regulation of icam-1 in polymicrobial sepsis. J Surg Res.

[11] Kragsbjerg P, Holmberg H, Vikerfors T (1995). Serum concentrations of interleukin-6, tumour necrosis factor-alpha, and C-reactive protein in patients undergoing major operations. Eur J Surg.

[12] Nishioku T, Dohgu S, Takata F, Eto T, Ishikawa N, Kodama KB, Nakagawa S, Yamauchi A, Kataoka Y (2009). Detachment of brain pericytes from the basal lamina is involved in disruption of the blood-brain barrier caused by lipopolysaccharide-induced sepsis in mice. Cell Mol Neurobiol.

[13] Iwashyna TJ, Ely EW, Smith DM, Langa KM (2010). Long-term cognitive impairment and functional disability among survivors of severe sepsis. JAMA.

[14] van den Boogaard M, Schoonhoven L, Evers AW, van der Hoeven JG, van Achterberg T, Pickkers P (2012). Delirium in critically ill patients: impact on long-term health-related quality of life and cognitive functioning. Crit Care Med.

[15] Brummel NE, Jackson JC, Pandharipande PP, Thompson JL, Shintani AK, Dittus RS, Gill TM, Bernard GR, Ely EW, Girard TD (2014). Delirium in the ICU and subsequent long-term disability among survivors of mechanical ventilation. Crit Care Med.

[16] Pandharipande PP, Girard TD, Jackson JC, Morandi A, Thompson JL, Pun BT, Brummel NE, Hughes CG, Vasilevskis EE, Shintani AK (2013). Long-term cognitive impairment after critical illness. N Engl J Med.

[17] Liu H, Li J, Guo J, Shi Y, Wang L (2022). A prediction nomogram for neonatal acute respiratory distress syndrome in late-preterm infants and full-term infants: a retrospective study. EClinicalMedicine.

[18] Fan T, Wang H, Wang J, Wang W, Guan H, Zhang C (2021). Nomogram to predict the risk of acute kidney injury in patients with diabetic ketoacidosis: an analysis of the MIMIC-III database. BMC Endocr Disord.

[19] Vickers AJ, Cronin AM, Elkin EB, Gonen M (2008). Extensions to decision curve analysis, a novel method for evaluating diagnostic tests, prediction models and molecular markers. BMC Med Inform Decis Mak.

[20] Guenther U, Theuerkauf N, Frommann I, Brimmers K, Malik R, Stori S, Scheidemann M, Putensen C, Popp J (2013). Predisposing and precipitating factors of delirium after cardiac surgery: a prospective observational cohort study. Ann Surg.

[21] Perry VH, Cunningham C, Holmes C (2007). Systemic infections and inflammation affect chronic neurodegeneration. Nat Rev Immunol.

[22] Murray C, Sanderson DJ, Barkus C, Deacon RM, Rawlins JN, Bannerman DM, Cunningham C (2012). Systemic inflammation induces acute working memory deficits in the primed brain: relevance for delirium. Neurobiol Aging.

[23] Widmann CN, Heneka MT (2014). Long-term cerebral consequences of sepsis. Lancet Neurol.

[24] Tauber SC, Djukic M, Gossner J, Eiffert H, Bruck W, Nau R (2021). Sepsis-associated encephalopathy and septic encephalitis: an update. Expert Rev Anti Infect Ther.

[25] Luetz A, Heymann A, Radtke FM, Chenitir C, Neuhaus U, Nachtigall I, von Dossow V, Marz S, Eggers V, Heinz A (2010). Different assessment tools for intensive care unit delirium: which score to use?. Crit Care Med.

[26] Ely EW, Margolin R, Francis J, May L, Truman B, Dittus R, Speroff T, Gautam S, Bernard GR, Inouye SK (2001). Evaluation of delirium in critically ill patients: validation of the confusion Assessment Method for the Intensive Care Unit (CAM-ICU). Crit Care Med.

[27] Radtke FM, Franck M, Oppermann S, Lutz A, Seeling M, Heymann A, Kleinwachter R, Kork F, Skrobik Y, Spies CD (2009). [The Intensive Care Delirium Screening Checklist (ICDSC)--translation and validation of intensive care delirium checklist in accordance with guidelines]. Anasthesiol Intensivmed Notfallmed Schmerzther.

[28] Bergeron N, Dubois MJ, Dumont M, Dial S, Skrobik Y (2001). Intensive care Delirium Screening Checklist: evaluation of a new screening tool. Intensive Care Med.

[29] Qin L, Wu X, Block ML, Liu Y, Breese GR, Hong JS, Knapp DJ, Crews FT (2007). Systemic LPS causes chronic neuroinflammation and progressive neurodegeneration. Glia.

[30] Cardona AE, Li M, Liu L, Savarin C, Ransohoff RM (2008). Chemokines in and out of the central nervous system: much more than chemotaxis and inflammation. J Leukoc Biol.

[31] Pinto SS, Gottfried C, Mendez A, Goncalves D, Karl J, Goncalves CA, Wofchuk S, Rodnight R (2000). Immunocontent and secretion of S100B in astrocyte cultures from different brain regions in relation to morphology. FEBS Lett.

[32] Michels M, Michelon C, Damasio D, Vitali AM, Ritter C, Dal-Pizzol F (2019). Biomarker predictors of Delirium in acutely ill patients: a systematic review. J Geriatr Psychiatry Neurol.

[33] Matsuda J, Kato S, Yano H, Nitta G, Kono T, Ikenouchi T, Murata K, Kanoh M, Inamura Y, Takamiya T (2020). The sequential organ failure Assessment (SOFA) score predicts mortality and neurological outcome in patients with post-cardiac arrest syndrome. J Cardiol.

[34] Kurtz P, Taccone FS, Bozza FA, Bastos LSL, Righy C, Goncalves B, Turon R, Machado MM, Maia M, Ferez MA (2021). Systemic severity and organ dysfunction in subarachnoid hemorrhage: a large Retrospective Multicenter Cohort Study. Neurocrit Care.

[35] Dellinger RP, Levy MM, Rhodes A, Annane D, Gerlach H, Opal SM, Sevransky JE, Sprung CL, Douglas IS, Jaeschke R (2013). Surviving sepsis campaign: international guidelines for management of severe sepsis and septic shock: 2012. Crit Care Med.

[36] Smith JC, Abdala AP, Borgmann A, Rybak IA, Paton JF (2013). Brainstem respiratory networks: building blocks and microcircuits. Trends Neurosci.

[37] Del Negro CA, Funk GD, Feldman JL (2018). Breathing matters. Nat Rev Neurosci.

[38] Jackson JC, Girard TD, Gordon SM, Thompson JL, Shintani AK, Thomason JW, Pun BT, Canonico AE, Dunn JG, Bernard GR (2010). Long-term cognitive and psychological outcomes in the awakening and breathing controlled trial. Am J Respir Crit Care Med.

[39] Houwink AP, Rijkenberg S, Bosman RJ, van der Voort PH (2016). The association between lactate, mean arterial pressure, central venous oxygen saturation and peripheral temperature and mortality in severe sepsis: a retrospective cohort analysis. Crit Care.

[40] Ryoo SM, Lee J, Lee YS, Lee JH, Lim KS, Huh JW, Hong SB, Lim CM, Koh Y, Kim WY (2018). Lactate Level Versus Lactate Clearance for Predicting Mortality in patients with septic shock defined by Sepsis-3. Crit Care Med.

[41] Sakpichaisakul K, Supapannachart KJ, El DM, Szakmar E, Yang E, Walsh BH, Robinson JN, Cherkerzian S, Volpe JJ, Inder TE (2021). Blood gas measures as predictors for neonatal encephalopathy severity. J Perinatol.

[42] Skare C, Karlsen H, Strand-Amundsen RJ, Eriksen M, Skulberg VM, Sunde K, Tonnessen TI, Olasveengen TM (2021). Cerebral perfusion and metabolism with mean arterial pressure 90 vs. 60 mmHg in a porcine post cardiac arrest model with and without targeted temperature management. Resuscitation.

[43] Peacock M (2021). Phosphate metabolism in Health and Disease. Calcif Tissue Int.

[44] Li Z, Shen T, Han Y (2022). Effect of serum phosphate on the prognosis of septic patients: a retrospective study based on MIMIC-IV Database. Front Med (Lausanne).

[45] Rroji M, Figurek A, Viggiano D, Capasso G, Spasovski G. Phosphate in the context of cognitive impairment and other neurological Disorders occurrence in chronic kidney disease. Int J Mol Sci 2022, 23(13). 10.3390/ijms23137362 PMC9266940 35806367

[46] Reis JP, Launer LJ, Terry JG, Loria CM, Zeki Al Hazzouri A, Sidney S, Yaffe K, Jacobs DR, Whitlow CT, Zhu N (2013). Subclinical atherosclerotic calcification and cognitive functioning in middle-aged adults: the CARDIA study. Atherosclerosis.

[47] Slinin Y, Vo T, Taylor BC, Murray AM, Schousboe J, Langsetmo L, Ensrud K (2018). Osteoporotic fractures in men study G: serum phosphate and cognitive function in older men. Int J Geriatr Psychiatry.

